# An AIDS-Denialist Online Community on a Russian Social Networking Service: Patterns of Interactions With Newcomers and Rhetorical Strategies of Persuasion

**DOI:** 10.2196/jmir.3338

**Published:** 2014-11-17

**Authors:** Peter Meylakhs, Yuri Rykov, Olessia Koltsova, Sergey Koltsov

**Affiliations:** ^1^National Research University Higher School of EconomicsLaboratory for Internet StudiesSt. PetersburgRussian Federation

**Keywords:** consumer health information, Internet, online communities, AIDS denialists, quality of health information on the Internet, netnography, qualitative research

## Abstract

**Background:**

The rise of social media proved to be a fertile ground for the expansion of the acquired immune deficiency syndrome (AIDS)-denialist movement (in the form of online communities). While there is substantial literature devoted to disproving AIDS-denialist views, there is a lack of studies exploring AIDS-denialists online communities that interact with an external environment.

**Objective:**

We explored three research areas: (1) reasons for newcomers to come to an AIDS-denialist community, (2) the patterns of interactions of the community with the newcomers, and (3) rhetorical strategies that denialists use for persuasion in the veracity of their views.

**Methods:**

We studied the largest AIDS-denialist community on one of the most popular social networking services in Russia. We used netnography as a method for collecting data for qualitative analysis and observed the community for 9 months (at least 2-3 times a week). While doing netnography, we periodically downloaded community discussions. In total, we downloaded 4821 posts and comments for analysis. Grounded theory approach was used for data analysis.

**Results:**

Most users came to the community for the following reasons: their stories did not fit the unitary picture of AIDS disease progression translated by popular medical discourse, health problems, concern about HIV-positive tests, and desire to dissuade community members from false AIDS beliefs. On the basis of strength in AIDS-denialist beliefs, we constructed a typology of the newcomers consisting of three ideal-typical groups: (1) convinced: those who already had become denialists before coming to the group, (2) doubters: those who were undecided about the truth of either human immunodeficiency virus (HIV) science theory or AIDS-denialist theory, and (3) orthodox: those who openly held HIV science views. Reception of a newcomer mainly depended on the newcomer’s belief status. Reception was very warm for the convinced, cold or slightly hostile for the doubters, and extremely hostile or derisive for the orthodox. We identified seven main rhetorical strategies of persuasion used by the denialists on the “undecided”.

**Conclusions:**

Contrary to the widespread public health depiction of AIDS denialists as totally irrational, our study suggests that some of those who become AIDS denialists have sufficiently reasonable grounds to suspect that “something is wrong” with scientific theory, because their personal experience contradicts the unitary picture of AIDS disease progression. Odd and inexplicable practices of some AIDS centers only fuel these people’s suspicions. We can conclude that public health practitioners’ practices may play a role in generating AIDS-denialist sentiments. In interactions with the newcomers, the experienced community members highlighted the importance of personal autonomy and freedom of choice in decision making consistent with the consumerist ideology of health care. The study findings suggest that health care workers should change a one-size-fits-all mode of counseling for a more complex and patient-tailored approach, allowing for diversity of disease progression scenarios and scientific uncertainty.

## Introduction

### Background

The rising role of new social media in the field of health can adequately be described as a “double-edged sword”. On one hand, new social media can deliver patients empowerment in doctor-patient relationships and be a medium of new evidence-based eHealth interventions and a platform for patient online support communities where they can share useful practical experience on coping with a chronic disease. In short, social media can help create a new type of patient, the ePatient, who is “equipped, enabled, empowered, and engaged in their health and health care decisions” [1]. In contrast, critics of techno-enthusiasts who embrace this new form of communication point out that the Internet in general, and new social media in particular, can help spread pernicious, antiscientific views on health (eg, social acceptance of anorexia [2] or anti-vaccination movement views [3-5]). Thus, the dubious or downright pernicious quality of some of the information circulating on the Internet has rightly been named as a major concern for eHealth [6], and for medicine as a whole as well [7].

### AIDS-Denialist Movement

While some of these antimedicine movements, such as the antivaccination movement, have been the object of extensive scientific research [3-5,8,9], the acquired immune deficiency syndrome (AIDS)-denialist movement has received little attention from social science despite its having been a focus of huge public controversies and a long-standing cause of trouble for medical and activist communities dealing with HIV/AIDS [10,11]. The “AIDS-dissident movement” as they call themselves denies either human immunodeficiency virus (HIV) existence or a connection between HIV and AIDS. The majority of the existing research (eg, [12-16]) is devoted to the analysis of the situation in South Africa where in the late 1990s and early 2000s then-president Mbeki banned use of antiretroviral therapy (ART) used for HIV treatment in state hospitals, which by some estimates resulted in more than 300,000 AIDS-related deaths and hundreds of thousands of new infections [17]. The other large part of the research to date is dedicated to disproving AIDS-denialists views as unscientific (one of the latest and brilliant examples of such studies is [18]). Few studies examine the AIDS-denialist movement as a *movement* and not just a system of *views* (with a notable exception of Kalichman’s [19] and Nattrass’s [20] studies), and to date we are not aware of a single study that explores this movement in Russia or Former Soviet Union (FSU) countries. This is deplorable as the AIDS-denialist movement is alive and well in these countries. While it is difficult to determine the exact extent of the AIDS-denialist movement’s influence on public health, some studies indicate that it is significant in some communities. A survey at minority gay pride events in four American cities in 2005 found that around one third of attendees doubted that HIV caused AIDS [21]. A survey of people living with HIV (PLWH) of African-American background conducted by Kalichman et al [22] showed that one in five participants believed that there is no proof that HIV causes AIDS and that HIV medicines do more harm than good. AIDS denialism has proved to have a negative impact on those who endorse it. Thus, in the same study it was found that holding denialist beliefs about AIDS was related to refusing HIV treatments and poor health outcomes. AIDS conspiracy theories (see [23] for review of available evidence on AIDS conspiracy beliefs among African Americans) are also an obstacle for HIV prevention and treatment [22,24-26].

The rise and global penetration of the Internet has opened a large window of opportunities for AIDS denialists, who quickly jumped on the bandwagon. As the pro-denialist Group for the Scientific Reappraisal of the HIV/AIDS Hypothesis (“Reappraising AIDS”) wrote on its website: “Thanks to the ascendance of the Internet, we are now able to reinvigorate our informational campaign” (quoted in [10]). The works of Kalichman et al [22], Smith and Novella [10], Nattrass [11], and other scholars underscore the role of the Internet in dissemination of AIDS-denialist misinformation.

Although there are no reliable data on the influence of the AIDS-denialist movement in Russia and FSU countries (either online or offline), Russian PLWH community leaders both in public talks and in informal talks with the current project team members admit that the AIDS-denialist movement is on the rise and that proliferation of social networking services (SNS) in FSU countries contributes to its growing influence. As a leader of one of the most prominent PLWH communities in Russia (who regularly monitors AIDS-denialist activity on the Internet) put it, “we are losing the battle [with AIDS denialists] on the Internet”.

To summarize, the growing presence of the AIDS-denialist movement on SNS presents a serious public health threat, which contributes to higher morbidity and mortality from AIDS and HIV-related diseases, and further spread of HIV among the populace. All this warrants research of the AIDS-denialist movement on the Internet in general, and on the social networking services in particular. To our knowledge, this is the first study of its kind.

### Study Objectives

This is an explorative study of the most numerous AIDS-denialist online communities on one of the most popular social networking services in Russia (its users also include millions of people from other FSU countries). As the spread of denialists’ views and the recruitment of new members into the movement are particularly challenging for public health, we have decided to (1) examine the reasons people come to the group, (2) analyze how the community deals with the newcomers, and (3) describe rhetorical strategies employed by deniers for persuasion in the veracity of their views. Gaining such knowledge could be of significant value for designing Internet interventions directed at counteracting the influence of AIDS denialists on the Internet.

## Methods

### Object of Study

We have chosen an SNS group (like Facebook groups) with a manifestly AIDS-denialist name, which is open for everybody who is willing to join. By community members, we can mean only those who have formally signed up for the SNS group: in the broadest “actionist” sense, those who participate in its activities regardless of formal belonging to the group; and finally, in the most restrictive sense, only those who formally belong to the community and also participate in its activities. We used the third definition when we drew a map of friendship in the group and the second when we calculated some statistics on activities in the community and for a qualitative analysis of the community posts and comments.

The group is highly visible to people who seek information about HIV and AIDS. Thus, searching the word “HIV” in Russian (at the moment of this paper’s submission) using Google’s search engine (the second most popular engine among Russian users—33.9% of users [27]) returns results that include the group’s name in the top ten list. Similar results are generated from searching “AIDS” on Google and searching either of these words on the SNS search engine. In turn, the group’s high visibility on search engines leads to a higher probability that users seeking information on HIV and AIDS will click the group’s hyperlink (according to the findings of Eysenbach and Kohler [28], online health information seekers inspect the first 10 search results 97.2% of the time).

During the project’s execution (March to November 2013), the group numbered around 13,000 members and had existed for almost 5 years, with the date of the first post being December 8, 2008. The primary group’s mission statements listed on its webpage are “saving peoples’ lives” from the “AIDS industry” and spreading the true word about the AIDS conspiracy. The group contains 21 hyperlinks, the majority of which are other AIDS-denialist groups or their websites on the Internet, other antimedicine groups such as “Vaccines kill”, and nationalistic groups. The group lists nine moderators who have played a crucial role in its functioning by heavily moderating its content. Like all groups in the studied SNS, the group contains a message board called “the wall”, the most visible discussion space that has become the main object of analysis. The “wall” is where most newcomers come and it is also the place where the most heated discussions take place—apparently because it is the best place to post for attracting attention. Besides that, the group site contains 104 documents, hundreds of videos, and 284 “themes” (ie, discussion threads that vary in length from one to thousands of posts). Videos mostly include pro-denialist ads, various news items, or heavily criticized antidenialist materials. Most are re-posted from regular media and YouTube, some of which were made in Russian, with others dubbed by the sources from which they were borrowed. The main topics in discussion threads include “scientific” justifications of AIDS-denialist assertions, legal advice, discussion of AIDS in terms of conspiracy theory, advice on how to deal with medical institutions, advice for pregnant women, “harm and consequences of ART”, and direct advice topics such as “Don’t test for HIV!”. While antidenialist activists claim that such direct calls contradict Russia’s HIV laws, Russian legislation does not directly prohibit dissemination of false medical information.

### Data Collection

For outlining the group’s general picture, we used VKminer, a software developed in our lab that helped us map the friendships in the group. With this program, we also were able to download the content of the wall for the entire 2-year period and count the number of posts, comments, and likes for every participant in the “wall” activities.

We used netnography as a method for collecting data for qualitative analysis. Netnography is “a specialized form of ethnography adapted to include the unique computer-mediated contingencies of today’s social worlds” [29]. To put it more simply, netnography is ethnography on the Internet, which means that the observations are not quantified but analyzed in-depth using various qualitative data analysis methods. We observed the community during 9 months (at least 2-3 times a week and sometimes more frequently if there was “something up” in the community like a scandal or extremely frequent posting and commenting). The group is highly moderated, so in our case netnography, which implies frequent periods of continuous presence in the field, turned out to be particularly useful. Some posts and comments (especially those that were written by adherents of the scientific theory of HIV) lived for an hour or less before moderators removed them. Thus, we downloaded posts immediately in many cases, as there was substantial risk that we would not see them the next time we visited the community. Unable to monitor 24/7, we lost some posts and comments but were able to guess the gist from other posters’ later comments. In total, we downloaded 4821 posts and comments for qualitative analysis.

### Data Analysis

Social network analysis with NodeXL was used for mapping the group’s “quantitative portrait”, while for analysis of qualitative data we used Grounded Theory approach [30]. We used freeware QDA package Open Code 4.01 for computerized qualitative data analysis. While doing netnography, some notions that could be coded already cropped up, so when we started coding we had the initial set of codes (and memos indicating some potentially fruitful directions). Some of these codes did not work out; for instance, we thought the question of homosexuality would be controversial and actively debated but we were wrong. Only some members expressed homophobic attitudes, and their posts did not generate substantial reaction from other online community members. Also, at the start of the project our research questions were too broad to use as codes. So we started open coding, that is, coding fragments of text relevant to the research questions. In so doing, we started seeing some patterns in the texts, which we had not thought of at the start of the project. Thus emergent codes become apparent while, as we noted, some old codes turned out to be fruitless. As a result, we succeeded at achieving conceptual saturation, when each category or theme relevant for our study was developed “fully in terms of its properties and dimensions” [30].

Some of the participants used their real names (or at least, positioned themselves this way), some fearing possible stigmatization used fictional names (and informed the community members about it), and some went by nicknames. We use pseudonyms in this paper when we present community members’ quotes.

## Results

### Community Structure and Participants’ Activity

**Figure 1:**
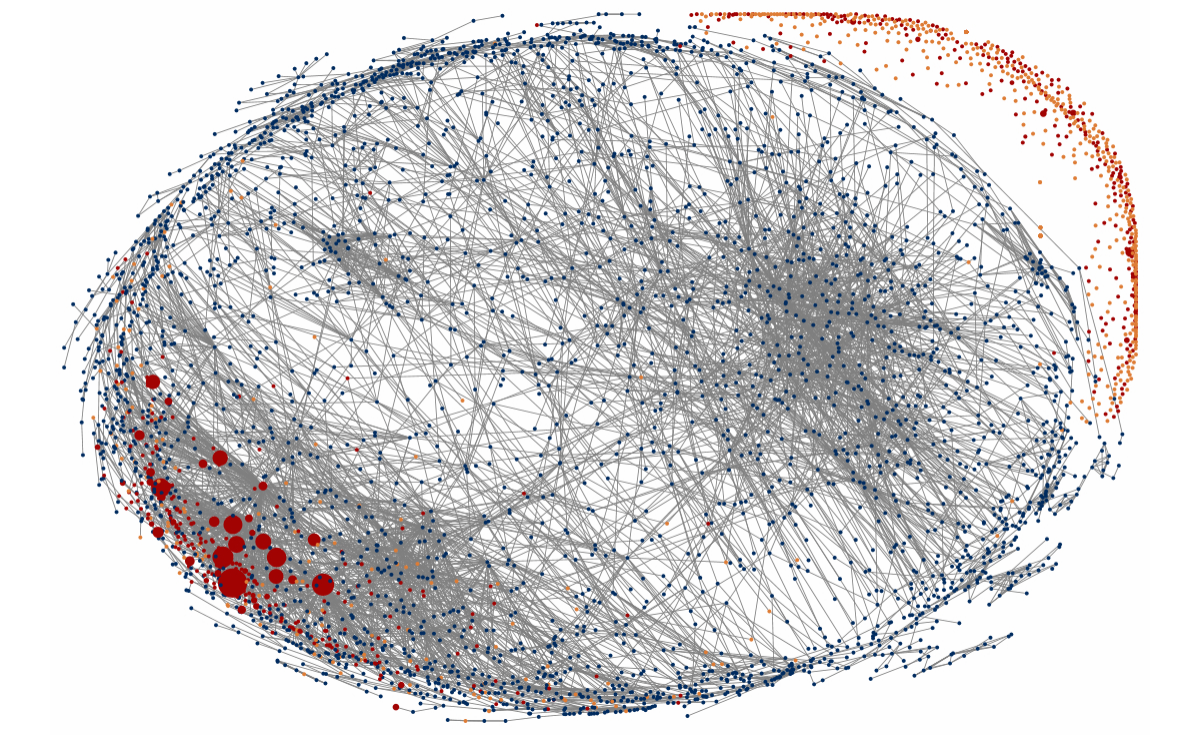
Friendship network of the community (red: participants who post and/or comment; orange: those who only give likes; blue: non-active members; non-active isolates excluded).

Analysis has shown that 61.31% (8051/13,131) of the group members were isolates, that is, they had no friends in the group. The majority (3425) of the non-isolated members belonged to the largest connected component, while the second largest component contained only 20 nodes. The visualization of the largest connected component and of the 700 active isolates is represented in Figure 1. By “active”, we mean the group members who participated in the group activity at least once in the form of a post, a comment, or a like. The size of nodes is proportional to the participants’ contribution to the content generation.

We found that the share of active online community members was 9.39% (1234/13,131). Only 4.32% (468/13,131) of group members generated content (posts or comments) while the rest, 5.07% (666/13,131), were only attention-givers in the form of “likes”. In sum, the community consists of a small core producing all the content and a large number of readers or potential readers, a small proportion of whom sometimes approves of what they read. Such community structure is not unique; however, it is not typical for inactive groups, where a dense core is seldom observed and the scarce activity is usually more evenly distributed among the moderately active members (unless such groups are “fanpages”). The dense core is therefore an indicator of intensive group dynamics and real communicative processes in the community.

### Reasons for Coming to the Group and Typology of Newcomers

Most people came to the community for the following reasons: their stories did not fit the general AIDS disease narrative (see below), curiosity, concern about HIV-positive tests, desire to dissuade the community members from false AIDS beliefs, or to support them in their struggle for truth. Many of the newcomers were in confusion and despair because of their diagnosis. Consider the following quote from a newcomer’s post that illustrates one of the most important reasons for coming to the group that we were able to pinpoint—a contradiction of the newcomer’s life story with her vision of the disease progression:

Here what I did when I was initiated into the caste of “the chosen”—I sought for anybody to talk about it openly and apart from psychological support counselor from AIDS-center I didn’t find anybody. After conversation with their psychologist I came to the conclusion I should get registered but for some reason someone inside told me: don’t rush…wait…My husband took the test and got a negative result! And from this moment the internal struggle began and soul-searching, and I would say God helped me to find you because before that I hadn’t even used [the name of SNS].

Olga

From this quote we can see that Olga started to doubt the “official theory” after she had learned that her husband was negative despite their having unprotected sexual relations, which, as she sees it, contradicts the HIV science theory according to which positives infect negatives. Another group member tells how her viral load has decreased “by itself” and her immune status rose, interpreting it as evidence of the fallacy of the “official theory”:

When I was pregnant I was diagnosed with HIV. And on the fifth month [of my pregnancy] my immune status is 350 cells – it is very little. it is thought that if less than 250, it is already AIDS. Viral load 85000...Next test: Immune status 750, viral load – 25000. That is, I did not take any medicines, and the viral load decreased by itself. According to the theory this is impossible. I asked them where did 60000 viruses go, one said “he doesn’t know”, the second that “maybe they mixed up something in the lab”…At this point I stopped coming to the AIDS-center.

Thus, from community members posts (old and new), we can see that their stories or lab tests results contradict (or seem to contradict) what we call the “AIDS-metanarrative”. Though each member writes about one or two contradictions with this metanarrative, combining our findings we can construct a schematic narrative, many elements of which are widely known to the public from popular and popular medical discourses. This metanarrative can be outlined as follows.

People get infected with HIV in situations of risk (such as needle sharing in injection drug use or unprotected sexual contact). Then in a certain period, their immune system (CD4 count) starts to lower and viral load starts to rise, and at a certain point their depleted immune system fails to defend them from a range of diseases and they die, unless they start taking highly active antiretroviral therapy (HAART). During the entire period of the disease progression, they are contagious and infect their sexual or injection partners. Pregnant women have a high chance of transmission of HIV to their children if they do not take antiretrovirals during pregnancy.

However, there are some points that contradict or seemingly contradict this AIDS-metanarrative that we saw in the community members’ posts: (1) absence of a risk situation: “I couldn’t get it because I have never used drugs or cheated on my partner, and I’m 100% sure that he didn’t cheat on me either”, (2) nontransmission of HIV from a positive to a negative: “I live with my husband and we have unprotected sex, and still seven years later he’s negative”, (3) nontransmission of HIV through sharing injection equipment: “My friend was a junkie and he shared needles with other junkies but he never caught HIV”, (4) lowering of the viral load without treatment: “My viral load dropped despite I faked taking HAART and threw out the pills”, (5) rise of the immune status without treatment: “My CD4 count rose even though I didn’t take HAART”, and (6) death of HIV-positives despite taking HAART: “People take HAART and die nevertheless”.

On the basis of strength in the AIDS-denialist beliefs, we have constructed a simple typology of the newcomers that consists of the three ideal-typical groups: (1) the convinced: those who already had become deniers before coming to the group, (2) the doubters: those who presented themselves as undecided as to the truth of either HIV science theory or AIDS-denialists theory, and also often posed uncomfortable questions that cast doubt over denialist views, and finally (3) the orthodox: those who openly held HIV science views.

### Patterns of Interactions Between the Group’s Experienced Members and the Newcomers

Reception of newcomers and the choice of a rhetorical strategy addressed to newcomers strongly depended on their presentation of self to the group. The decisive factor that determined the type of reception received by newcomers was, unsurprisingly, their “belief status” in the denialist views expressed in their post, although other factors such as confusion or self-confidence, cheerfulness, or a gloomy tone also mattered to some extent. Reaction of the group can be understood as positive, neutral, or negative depending on the comment’s sentiment to the newcomer’s post and also by the quantity of “likes” the post gets. Although we did not calculate “likes” formally for every newcomer’s post, the difference in likes between the “convinced” and the other types of newcomers is striking. While the “convinced” often got from 10-20 likes, all other newcomers got 0-3 likes. The content of the post with the highest chance of getting many likes was the “thank you” message to the community or/and expressed despisal of HAART. Consider the following post from Natalia that got 16 likes:

Hey guys, thank you for your community! I got “+” on the tenth week of my pregnancy. Husband “-”!!! I thank him that he didn’t turn his back on me, we together started to figure out what’s going on, to enter into details. In AC [AIDS-Center] they prescribed inviraza, kombivir, and ritonavir. Two pills of each medicine twice a day! It’s 12 pills a day!!!!! Holy shit! Considering that even when I have a banal cold I have never taken anything. AC worked all my nerves!!! After visiting them I had a stomach pain! Having read all your posts, having watched the videos I got convinced that all this is a big swindle. I won’t go to AC ever. The health of my child and my nerve cells are more important. P.S. I flushed the pills down the drain! Thank you so much!!!

But apart from likes, community members gave a verbal welcome in their comments to her post. She received emotional support and welcome messages, such as, “Welcome to the group!”, “Good luck and patience!”, and “Keep us informed about your battle with AC”. We call this strategy “rhetoric of reinforcement” as the newcomer is already convinced in AIDS-denialist views and the community only supports and reinforces the views and the feeling of community belonging.

A different situation arises with the doubters. Any degree of doubt in the truthfulness of denialists tenets almost always caused an ostentatiously cold or hostile reaction. Nadia drove this point home in her answer to Jenia, a “doubter”:

Jenia, I think that our group should be only for true dissidents, those, who have no qualms about their positions! And you create your own group for those, who’re neither here, nor there. And you will persuade each other, whether the virus exists. Let’s not interfere with each other. Sometimes one NEEDS necessary information—on lawyer’s advice, refusal [from medical treatment], and this information is just impossible to find in the mumbo-jumbo of those who still need to be persuaded and seek an answer to the question “whether the virus exists?”

By far the most popular answer that the doubters received to their questions was advice to “read the group’s materials”:

There are a lot of materials in the group. Please, understand us, we can’t answer the same questions every day, every day we are asked the same things, by familiarizing yourselves with the group materials you will understand who benefits and how, and there is a lot to benefit from.

Anya

Such refusals to answer the questions and reluctance to interact (which can be dubbed “strategy of avoiding”) met resistance from the doubters. Despite the cold reception and answer-dodging, the doubters tried to get answers to their questions by the following rhetorical devices: blaming the group, justification of their doubts, and appeal to the group’s mission. For instance, Alena blamed the group and simultaneously appealed to the group’s mission—spreading the word about HIV-conspiracy:

Is it so hard to copypaste the specific links?Oh yes, it’s much easier to write a derogatory message—10000 characters long—about the lack of intelligence of the one who’s asked the question. Just be forewarned—after such hospitable reception I (and other interested people) have a right to think about you whatever we want. If you want to be heard and understood, learn tact and respect to the interlocutor. Aggression is inappropriate in preaching.

The experienced group members to whom these requests and reproaches were addressed reacted in a defensive or even hostile manner. They explained their annoyance by denying allegations that they tried to “make” someone believe in anything, thereby appealing to the principle of “free choice”. Thus, Georgiy responded to Alena’s accusations: “This is not a place for preaching, and nobody makes you believe in anything. Actually, nobody owes you anything. You yourself choose what to believe in and whom to believe”. Alisa supported him:

Read the stories of people in the group. There are people here that have [been diagnosed with] HIV, and they have healthy children, don’t drink tera [ART], don’t infect their wives (husbands) —don’t these facts seem to you striking? And whether believe us—healthy people or people who are dying from tera and advocate tera at that—is up to you.

It is worth noting that newcomers often came to the community seeking advice, for instance, whether to take HIV test or not. Despite the experienced community members’ main advice of referral to the group materials, they also did give direct advice. It should be stressed that such advice in the overwhelming number of cases contradicted the seasoned community members’ position on freedom of choice and unwillingness to “enforce” their own point of view as this advice was clearly based on the denialist dogma. The most frequently observed advice that the newcomers received were “Don’t take HIV tests!”, “Don’t go to the AIDS-center!”, “Don’t take ART!”, “Don’t believe the HIV tests results!”, “Treat real diseases, not test results”, “Don’t succumb to stress as stress causes real diseases”, “Live on as though there was never HIV positive test, enjoy your life, you’re not sick with anything”, and “Live a healthy lifestyle and everything will be all right.”

Finally, the last type of newcomer according to our typology, the orthodox, come to the group page either out of curiosity or willing to persuade the community members in the falsehood of their beliefs. As we wrote above, the overwhelming number of the posts and comments written by the orthodox were swiftly removed, but the netnography method allowed capturing interactions of the hard-core deniers with the orthodox. In this case, deniers realized “strategies of protection”, and the orthodox were subjected, as a rule, to collective ridicule and insults. Marat responded to a newcomer, who presented himself as a doctor: “Kirill, the most amazing people are those, who got their education and still continue to push this HIV/AIDS scam, or some are ready to sell their souls for the money???”

Despite the experienced community members’ reluctance to interact with the newcomers who were doubters, the latter often succeeded in dragging the former into conversations and overcoming the “strategy of avoiding”. In this case, dedicated denialists exercised various rhetorical strategies in order to defend their position and simultaneously try to persuade doubters of the truthfulness of their ideas. Use of rhetorical strategies (ie, “strategies of persuasion”) with the doubters and unwillingness to interact with them may sound like a contradiction, but we should be aware that these strategies are addressed not only to a particular doubter but to the wider audience—all those who watch these interactions without engaging in them. (According to the group’s statistics, the daily average number of unique visitors from May 15 to June 15, 2013, was 381). Having analyzed and combined the denialists’ arguments scattered on different discussions and disputes on the wall, we have determined the main rhetorical strategies of persuasion.

### Denialists’ Rhetorical Strategies of Persuasion

#### Scientific Arguments

Denialists present the scientific community as having no proof of HIV existence, and the evidence produced by scientists as unconvincing or fabricated. However, in the modern world it takes science to disprove science. This is why denialists engage in “selective distrust of scientific authority” [10], that is, discarding the findings on HIV/AIDS that are agreed on in the scientific community, but putting forth what Nattrass calls “hero scientists” [20] who have evidence against this “concoction” but are silenced by those who take part in the global conspiracy (see below). Duesberg was by far the most popular “hero-scientist” referred to by the community members; others mentioned by the community members most frequently were Nobel prize winner Kary Mullis, and two Russian medical professionals—general practitioner Irina Sazonova and autopsist Vladimir Ageiyev. It should be noted that none of the Russian hero-scientists possesses credentials comparable to those of Duesberg and Mullis.

#### Ideological Arguments

Denialists claim that the myth of HIV appeared as a result of a global conspiracy between a secret world government and “Big Pharma”, who enforced the acceptance of this myth first in the United States and then in all other countries.

#### Underscoring the Importance of Personal Experience and Critical Thinking Compared to Unreflective Acceptance of Abstract Medical Knowledge

Thus, Alla wrote to Kirill, who presented himself as a doctor: “There are people here, who came to these conclusions [HIV is a myth] not on the basis of propaganda but on the basis of PERSONAL experience”. As we have shown earlier, this personal experience in many cases contradicts the dominant AIDS-metanarrative. This strategy is similar to the one described by Nattrass—the use of “living icons” [20] (ie, people living with “attributed” HIV diagnosis for prolonged periods seemingly without developing the disease symptoms) as the living proof of AIDS science’s fallacy (the most famous example being Christine Maggiore, an AIDS denialist who eventually died from AIDS). In our case, however, the living proof is not an AIDS-denialist celebrity from abroad but a regular person, that is, a group member who is present, thus making the denialist cause seem closer and more personal.

#### Underlining Material Interest of “Aidsologists”, Who Aim to Sell as Many Pills as Possible, Compared to the Denialists’ Lack of Material Interests

Cui bono argumentation is frequently used by AIDS-denialists, who constantly reiterate that they have no financial stake in the issue as opposed to “Aidsologists’ who are materially interested in propagating the “AIDS-myth.”

#### Pointing Out Suspicious Practices of AIDS Centers

AIDS centers’ specialists obscure, which for denialists means that they have something to hide. They do not give health records and test results to the patients but read these results to them instead. Indeed, in many Russian AIDS centers, patients’ health records and tests results are not given to them, which generates suspicion on the part of some patients. Denialists interpret these practices as the evidence of doctors’ participation in the global conspiracy.

#### Claims About Uselessness and Toxicity of Antiretroviral Therapy

One community member, Luda, writes “HIV is a pseudoscientific terrorism. People die from...drugs or poisonous therapy they receive”.

#### Use of “Morphed Science”

Morphed science (unconnected statements from legitimate sources taken out of context that are dispersed throughout the text) is used, as well as an abundance of highly technical jargon or as Kalichman calls it “technobabble” [19]. Kalichman writes about the purpose of this strategy: “Even scientifically trained readers will get lost in the illogic of morphed science. Morphed science can convince the untrained reader that the author is knowledgeable about AIDS while not understanding a word of what they are saying…The objective of technobabble in denialism is to present a façade of science within which it is easy to lose track of the details. Like morphing science, the goal is that readers render the material credible even if utterly unintelligible” [19].

### Doubter Reactions to Strategies

We would like to conclude this section by describing the doubters’ reactions to rhetorical strategies of persuasion directed to them. In most cases, they remained undecided as illustrated by the following quote from newcomer-doubter Semen: “There is no point to delve into this heap of articles and video materials, as both sides have a lot of evidence”. We found only a few cases when a doubter thanked the group for clarifying the issue and dispelling doubts as to the veracity of the denialist tenets. However, we cannot conclude based on these findings that denialists’ rhetorical strategies are ineffective, after all, many experienced group members wrote that initially they themselves had had doubts that were later dispelled as they obtained deeper knowledge of denialist evidence. As to answering the question of how these rhetorical strategies affect “lurkers”, a study with a totally different design is required. We observed only a single case when after interaction with the community’s experienced members, a newcomer-doubter took a pro-scientific stance on HIV.

## Discussion

### Principal Findings

Contrary to the widespread public health depictions of AIDS denialists as “crazy”, “delusional”, or insulated from reason by psychological “denial” [19], our study suggests that some of those who become AIDS deniers have sufficiently reasonable grounds to suspect that “something is wrong” with the orthodox theory. This is mostly because their personal experience contradicts the AIDS-metanarrative taken from medical and popular discourses, and it is commonly considered to be quite reasonable to have doubts when empirical facts do not fit the theory. Admittedly, not everybody would reject the medical and scientific knowledge on the basis of some facts that do not seem to fit in commonly held theories. Other factors influencing people to become AIDS denialists are obviously in play (psychological traits and trust in medical institutions will probably be some of them), but to portray these people as utterly irrational would be equally fallacious. Of course, this contradiction occurs not because the scientific theory of AIDS is wrong but because the AIDS-metanarrative is an oversimplified form of this theory leaving no room for different disease progression scenarios and scientific uncertainty. Smith and Novella wrote to this effect: “Oversimplifying AIDS science to the public lends itself to exploitation by AIDS deniers who remain ‘alive and well’ years after diagnosis with HIV. Yet the reality behind the scenes is often quite different. Every medical field has its legitimate controversies and complexities, and the process of science is often messy. Denial groups exploit the gap between public education and scientific reality” [10]. Odd and inexplicable (at least from the patients’ perspective) practices of some AIDS centers exploited by AIDS deniers for their own purposes only fuel suspicions of people who face this gap. Concordant to Blume’s [31] study of antivaccination movement, we can conclude that public health practitioners’ practices may play a role in generating AIDS-denialists’ sentiments.

We do not try to assert that understanding the multifaceted phenomenon of why some people become denialists can be achieved purely by analyzing their rational reasons for accepting denialist views. Obviously, there are deep psychological reasons for that. A well-known psychological concept of “being in denial” about one’s illness that brings both psychological (tranquility) and practical (one does need not to embark on a complex regimen of pill-taking) benefits is certainly a major factor in many cases of the denialist views. However, in this paper we tried to shift the focus from a traditional perspective of analyzing people’s psychological traits and their proclivity to being in denial to the question of social-structural production of denialism.

On a practical level, this means that in consultation with patients, practitioners should change a one-size-fits-all mode of counseling (the AIDS-metanarrative telling) to a more complex and patient-tailored approach, allowing for diversity of disease progression scenarios and open admission of scientific uncertainty on some HIV issues (when necessary) with concomitant emphasis that diversity and uncertainty do not undermine the basic principles and findings of HIV science. Elimination of the AIDS centers’ “shadow practices” could also be very helpful in building and/or sustaining trust in doctor-patients relationships and dispelling the conspiracy myth propagated by AIDS denialists.

Studying interactions of the experienced community members with the newcomers, we have also seen that the former do not try to recruit the latter by any means necessary (contrary to religious cults, eg, [32,33]) but instead highlight the importance of personal autonomy, critical thinking, and freedom of choice in decision making (again the picture that contradicts the familiar portrait of AIDS denialists as irrational fanatics). This finding is in accordance with the popular, or even dominant, consumerist ideology of health care, according to which patients are informed consumers that critically assess medical advice before accepting or rejecting it [3,31,34,35]. As Blume wrote about consumerist ideology in the case of the vaccination opponents: “As citizens, we were increasingly encouraged to think of ourselves as critical consumers, taking responsibility for our own health. Consumers, informed and empowered, have the right of choice…so why not here? Isn’t a critical stance towards vaccination, and hence the possibility of alternative viewpoints, a logical consequence of this ideological shift?” [31]. We may observe the same logic with the AIDS denialists. While patients’ growing power in modern health care is certainly a laudable and useful phenomenon [36], it has its downside—the erosion of trust in medical and scientific institutions in general, and consequently, adoption of antiscientific and destructive views. The AIDS-denialist movement bears witness to this.

This is not to say that the AIDS-denialist community is not interested in recruiting new members. We saw that they provide emotional support to the “convinced” type of the newcomers. In addition, we know that, though without enthusiasm (the lack of which can probably be attributed to fatigue of the experienced members of constantly answering the same questions), denialists employ rhetorical strategies of persuasion, which target not only the doubters but undecided lurkers as well.

We have also seen that members of the AIDS-denialist online community are not a homogenous group as they vary in the extent of their involvement in the group activities and in their belief status in denialist tenets. Further research is needed in order to address the issue of stratification among AIDS-denialist communities. While there is little use debating with hard-core denialists, we suggest spending time and resources on the doubters who have doubts both in the HIV science and the denialist views. Social network analysis methods could be particularly useful for determining “susceptibles” (similar to what is done for identifying susceptibles in other fields [37-40]) with regards to which Internet interventions designed to combat denialist views could be effective and efficient.

### Future Considerations

Comparison of denialist rhetorical strategies of persuasion identified in this paper with rhetorical strategies of the antivaccination movement [3,31,41,42] reveals considerable similarity. Toxicity of science-based treatment, sagas of brave scientists challenging medical orthodoxy, and other rhetorical devices that are employed in both movements are all cases in point. A comparative project addressing the issues of similarity and difference between these movements (and other antiscientific movements) would allow us to discern specific features of each movement and to understand what these movements have in common in terms of sociodemographic characteristics of their participants, interpretative frames, modes of action, and collective identities.

Finally, we need to gain deeper insight into why some HIV-positive people become AIDS denialists. Although we have received some preliminary answers to this question based on qualitative analysis of posts in this research, more work needs to be done. In-depth biographical interviews with HIV-positive AIDS-denialist movement members would certainly shed light on this question. Understanding the factors influencing adoption of denialist views could be very useful for practical efforts to combat the spread of AIDS-denialist sentiments.

## Abbreviations

AC: AIDS Center
AIDS: acquired immune deficiency syndrome
ART: antiretroviral therapy
HAART: highly active antiretroviral therapy
HIV: human immunodeficiency virus
PLWH: people living with HIV
QDA: qualitative data analysis
SNS: social networking services
